# Mixed-Methods Evaluation of the Delivery of Cancer Care to Teenagers and Young Adults in England and Wales: BRIGHTLIGHT_2021

**DOI:** 10.3390/curroncol33040211

**Published:** 2026-04-10

**Authors:** Rachel M. Taylor, Elysse Bautista-Gonzalez, Julie A. Barber, Jamie Cargill, Rozalia Dobrogowska, Richard G. Feltbower, Laura Haddad, Nicolas Hall, Maria Lawal, Martin G. McCabe, Sophie Moniz, Louise Soanes, Dan P. Stark, Bethany Wickramasinghe, Cecilia Vindrola-Padros, Lorna A. Fern

**Affiliations:** 1 Centre for Nurse, Midwife and AHP Led Research (CNMAR), University College London Hospitals NHS Foundation Trust, London NW1 2PG, UK; 2 Department of Statistical Science, University College London, London WC1E 6BT, UK; 3 Managed Service Network for Children and Young People with Cancer in Scotland, Kings Cross Hospital, Dundee DD3 8EA, UK; 4 Rapid Research Evaluation and Appraisal Lab (RREAL), Department of Targeted Intervention, University College London, London WC1E 6BT, UK; 5 Child Health Outcomes Research at Leeds, Leeds Institute for Data Analytics, School of Medicine, University of Leeds, Leeds LS2 9JT, UK; r.g.feltbower@leeds.ac.uk; 6 Patient Representative, BRIGHTLIGHT Young Advisory Panel, UK; 7 Division of Cancer Sciences, The University of Manchester, Manchester M13 9PL, UK; 8 Teenage Cancer Trust, London WC1V 7AA, UK; 9 Leeds Institute of Medical Research at St James’s, Leeds LS9 7TF, UK; d.p.stark@leeds.ac.uk; 10 Department of Targeted Intervention, University College London, London WC1E 6BT, UK; 11 Cancer Clinical Trials Unit, University College London Hospitals NHS Foundation Trust, London NW1 2PG, UK; lorna.fern@nhs.net

**Keywords:** cancer, young people, teenagers, young adults, BRIGHTLIGHT, coordination, collaboration, model of care, patient experience

## Abstract

Background: Healthcare policy in the United Kingdom recognizes that teenagers and young adults (TYAs: 16–24 years at diagnosis) require specialist care. In England, Principal Treatment Centers (PTCs) exist, delivering enhanced care exclusively within the PTC or as ‘joint care’ with designated hospitals (DHs). Central to this is the TYA multidisciplinary team (MDT) and an outreach model coordinating care between hospitals. We previously reported similar outcomes regardless of care location. Aims: To compare TYA experiences of care with healthcare professionals’ perspectives of the service they deliver. Methods: Mixed methods across England and Wales were used. The TYA-MDT identified TYAs who then received a postal invite to a cross-sectional survey capturing experiences of places of care, treatment, healthcare professional support (HCP), mental health, sexuality/fertility, clinical trials and care coordination. Comparisons were made based on exposure to care in a specialist TYA environment within 6 months of diagnosis: all-TYA-PTC (all care in the TYA-PTC, *n* = 70, 28%), no-TYA-PTC (no care in the TYA-PTC (*n* = 87, 35%): care delivered in a children/adult unit only), and joint care (care in a TYA-PTC and in a children’s/adult unit, *n* = 91, 36%). HCP perspectives were captured by rapid ethnography. Results: A total of 250/1056 (24%) TYAs participated. Overall, 200 (80%) rated their teams as excellent/good for helping them prepare for treatment. No evidence of significant differences existed between categories of care for proportions receiving support from key TYA-related professionals: TYA cancer nurse specialists (all-TYA-PTC *n* = 58, 91%; joint care *n* = 71, 88%; no-TYA-PTC *n* = 64, 82%) and social workers (all-TYA-PTC *n* = 30, 55%; joint care *n* = 36, 48%; no-TYA-PTC *n* = 28, 38%). A trend of diminishing support from youth support co-coordinators existed (all-TYA-PTC 63%; joint care 49%; no-TYA-PTC 40%, *p* = 0.069). This may explain why few differences in patient experiences existed across categories of care. Forty-nine HCPs participated. They were more critical in their interpretation of care, highlighting inequity in resources and challenges in some pathways and coordination. Conclusions: Similar access to age-appropriate support across care settings is likely to reflect recruitment methods. When TYAs are known to the MDT, age-appropriate care can be mobilized beyond TYA units, which could explain the equitable outcomes observed across different care locations in young people who responded to the survey. Nevertheless, gaps persist in communication and coordination, particularly within joint care models, and in the involvement of allied health professionals such as dieticians and physiotherapists, whose input is essential for rehabilitation and return to normal life. Strengthening these areas will require continued investment in workforce capacity and digital infrastructure to support genuinely coordinated, developmentally appropriate TYA cancer care.

## Simple Summary

Young people aged 16–24 with cancer need care that supports both their medical and emotional needs. In England, specialist hospitals called Principal Treatment Centers (PTCs) provide this, sometimes alongside local hospitals (“joint care”). Each PTC has a team of different types of professionals who try to make sure that young people get both medical treatment and emotional support. This is called the multidisciplinary team (MDT). Some young people will have all their care in a PTC, some will have no care in a PTC, and some young people will have ‘joint care’ in both a PTC and a normal cancer hospital. The MDT will try to make sure that young people not in the PTC also get support from the right professionals. This study aimed to compare the experiences of young people with cancer with the views of healthcare professionals. Surveys were sent to patients across England and Wales, and professionals took part in interviews. Of 1056 young people invited, 250 responded. We found that eight out of ten young people rated their care teams highly, and young people received similar rates of support from specialist nurses, youth support workers, and social workers across different care settings. Professionals, however, were more critical of the care they delivered, noting differences in resources and challenges in coordination. Overall, young people received similar levels of age-appropriate support, suggesting that when the MDT is involved, good care can be provided both within and outside specialist units. There are still gaps in how teams communicate and work together in joint care models, especially when involving key allied health professionals like dieticians and physiotherapists. Improving this will require more investment in staff and digital systems to deliver truly coordinated, age-appropriate cancer care for teenagers and young adults.

## 1. Introduction

Cancer care for teenagers and young adults (TYAs) aged 13–24 years was introduced in the United Kingdom (UK) in 1990 in response to outcomes for this age group being poorer than in children and older adults [1]. The model of care included an environment specific for TYAs, designed to be age-appropriate, and a workforce that understood the unique developmental challenges young people were going through with a cancer diagnosis [2]. Over the next 30 years, 28 TYA-specific units were developed across the UK. A key component of a model of TYA cancer care is integrated medical and psychosocial care, [3,4], which has been supported through the configuration of the workforce to include more specialist nurses, youth support coordinators, social workers and education mentors to address young people’s psychosocial needs. Most of these posts were funded by the third sector (Teenage Cancer Trust, Young Lives vs. Cancer, and Macmillan Cancer Support).

In 2005, this model of care was formally recognised in England through national guidance: the ‘Improving Outcomes Guidance (IOG) for Children and Young People’s Cancer’ [5]. Key recommendations in this guidance included provision of age-appropriate care, appropriately trained staff and the mandate that young people aged <19 years had to be treated in a TYA Principal Treatment Center (PTC), while those aged 19–24 were to be offered a choice of where to receive treatment but had ‘unhindered access to age-appropriate facilities and support’. It also recommended that care is delivered throughout the treatment pathway through a multidisciplinary team [5].

As there was limited evidence to underpin these recommendations, they were made based on expert professional opinion and TYA patient advocacy [6]. The National Cancer Action Team strove to implement the IOG recommendations, and by 2010, care had been semi-centralized to 13 TYA PTCs hosted in cities across England, ensuring there was access to age-appropriate care geographically. Acknowledging that travel and access to these PTCs may be difficult for some young people and their families, local hospitals were designated to deliver cancer care to provide some, if not all, of the recommendations in the IOG. The important central feature of these regional networks of care was the TYA MDT. Unlike tumour-specific MDTs, the TYA MDT was designed to address young people’s psychosocial and supportive care needs [7]. The model of care has therefore facilitated access to the age-appropriate aspect of care wherever young people are treated. As such, Scotland and Wales have similar models of care, and this is also being implemented in Northern Ireland [8,9,10].

Concurrent to the implementation of specialist TYA care, research was embedded from the onset to evaluate its benefit. Initially, this was provided through an ethnographic study of the first TYA unit in London, which showed the unit enabled a supportive bond for young people in an environment that was focused on their needs [11]. This environment and the TYA workforce were viewed as being more relaxed, accommodating normal routines and supportive of family’s needs. While there was cancer expertise, young people valued “the availability of an expert team of professionals with specialized insight into adolescents’ needs” [12]. Further supporting young people’s preferences for specialist TYA units, Denver conducted a mixed-methods study, where she found that young people without cancer expressed a more positive emotional response to TYA units rather than an adult cancer ward. This could not be attributed to any design feature, although the results indicated an overall impression of hospital space being more important than individual features. A subsequent focus group with young people with cancer noted that specialist units allowed them retain control and to maintain their personalities through identification with their surroundings. Being with other young people facilitated interaction and improved perceived levels of support [13].

The first national evaluation of the benefit of specialist care was presented by Birch, who showed that there was regional variation in young people accessing care, but those who accessed specialist care for central nervous system tumours, leukemia or lymphoma had better survival [14]. Survival benefits were further shown in a study in one region in the UK for young people with leukemia, who had a 73% risk of death if they were not treated in a TYA-PTC [15]. A subsequent evaluation from young people’s perspective was provided through the National Cancer Patient Experience Survey, a nationally administered survey to gather feedback from cancer patients on the quality of care they have received (https://www.ncpes.co.uk/). Overall, young people aged 16–24 years had poorer experiences of cancer care than the older adult cancer population. The comparison between those who were treated in a hospital with a specialist TYA unit to those who did not indicated better experiences in specialist care; however, responses suggested that improvements were still required to fulfil young people’s holistic needs [16]. While this showed promising support for specialist TYA care, this was a secondary analysis of data collected for government evaluation of services, which did not necessarily reflect services TYAs accessed, especially in the specialist units. It was also not designed to evaluate outcomes.

In 2012, BRIGHTLIGHT was launched as the national evaluation of TYA cancer services in England [17]. This was a programme of research, acknowledging that healthcare was too complex to capture benefit in a single study. It therefore focused on the environments where care was delivered [18,19], the workforce providing care [20,21], outcomes of care for young people and their caregivers [22,23,24,25], and an economic evaluation of the cost to young people and the National Health Service (NHS) [17]. BRIGHTLIGHT’s results indicated that outcomes were poorer if young people received care across multiple organizations, but this could have been due to the embryonic relationships in some networks. We surmised that these would have developed over the last decade and, therefore, outcomes would be improved [17]. This has largely been supported in BRIGHTLIGHT_2021, where we found no evidence of a difference in outcome based on where young people were treated [18]. This could be explained in part by the mechanisms implemented to increase collaboration and coordination of care [19]. To further evaluate TYA cancer care in England and Wales, this paper presents a comparison of young people’s experiences of care with healthcare professionals’ perspectives of the service they deliver.

## 2. Materials and Methods

### 2.1. Study Design

BRIGHTLIGHT_2021 was a convergent parallel mixed-methods study across England, Scotland and Wales, focusing on young people’s outcomes and experiences of care through a survey and healthcare professionals’ perspectives of how care was delivered through a rapid ethnography study. Data were collected between March 2022 and December 2023. The data presented in this paper has not been reported previously, although other aspects of the study have been reported [18,19].

### 2.2. Participants and Setting

Participants in both studies have been reported in detail elsewhere [18,19]. BRIGHTLIGHT_2021 recruited across England, Scotland and Wales. However, we were unable to collect data from professionals in Scotland; therefore, to examine like-for-like comparisons between young people and professionals, we have removed Scotland from this paper. In summary, young people were recruited in England and Wales through TYA MDTs, facilitating recruitment from across both nations. Young people needed to be aged 16–24 years at the time of a new cancer diagnosis and within 4 months of diagnosis. Exclusion criteria included young people whose end of life was imminent, had opted out of receiving information and were not able to receive a survey (in critical care or custodial care).

Healthcare professionals were identified by the TYA clinical lead in each region and were purposively sampled to represent professions within the MDT and the place they were delivering care (PTC or designated hospital).

### 2.3. Data Collection

The methods of data collection have been reported in detail elsewhere [18,19]. In summary, data collection from young people was through an adaptation of the BRIGHTLIGHT Survey, administered as a self-reported postal questionnaire with an online option. This contained five validated questionnaires measuring quality of life, health status, anxiety and depression, social support and illness perception. The results of this analysis have been reported previously; this paper focuses on the patient experience questions that explored places of care, treatment experiences, support from healthcare professionals, mental health, sexuality and fertility, access to clinical trials and coordination of care. Data from the Hospital Anxiety and Depression Scale (HADS) is reported here and is a measure of the presence and levels of depression and anxiety. It contains 14 items, which are answered on a four-grade Likert scale. Scores of 8–10 are defined as borderline, and scores of 11 and over are considered moderate/severe anxiety and depression [20,21]. Data collected from professionals was collected through semi-structured interviews informed by D’Amour’s conceptual framework on inter-organizational collaboration in healthcare networks. The interview schedule for this aspect of the study is available from Bautista-Gonzalez et al. [19].

### 2.4. Analysis

Survey data were analyzed using SPSS (29.0, IBM, Armonk, NY, USA), focusing on identifying trends and patterns in patient responses arising from the survey. Proportions were compared across groups using chi-square tests. A linear-by-linear association (chi-squared for trend) test was used to examine the relationship between support offered and received by the multidisciplinary team and categories of care (all-TYA-PTC, joint care, no-TYA-PTC). Comparisons were also made by age cohorts of 16–18 years and 19 years and above based on the current service specification for TYAs [22].

In parallel, data from the interviews were imported into NVivo (15, Lumivero, Denver, CO, USA) and analyzed using framework analysis [23]. The mixed-methods analysis involved triangulating the quantitative and qualitative analyses to explore the alignment of themes and variations in professionals’ perspectives on service delivery with young people’s survey responses. Additional supporting quotes were identified through content analysis of young people’s free-text comments to two questions: Was there anything particularly good about your NHS cancer care? Was there anything that could have been improved?

## 3. Results

A total of 1056 eligible young people from England and Wales were invited, 250 participated in the survey, and the response rate was 24% (Table 1). The majority were in the older TYA range (19–24 years, *n* = 161, 64%), female (*n* = 140, 56%) and white (*n* = 207, 83%). Fifty-five young people left free text in response to ‘was there anything particularly good about your NHS cancer care?’, 47 left comments in response to ‘was there anything that could have been improved?’ and 15 left free text under ‘other’.

A total of 49 professionals participated in the interviews from 11 sites (10 in England, and 1 in Wales). Participants included 18 medical doctors, 23 nurses, seven allied health professionals, four youth support coordinators and six from other professions. Forty-one were based in the TYA-PTC, six were based in designated hospitals and 11 worked across the regional network of the TYA-PTC.

The results from each dataset (young people’s survey responses and healthcare professional’s interviews) are presented in a joint display table for each theme so comparisons can be made. Where responses from young people do not total 250, this reflects not all young people answering the question.

### 3.1. Place of Care


**Young People**

**Figure 1:**
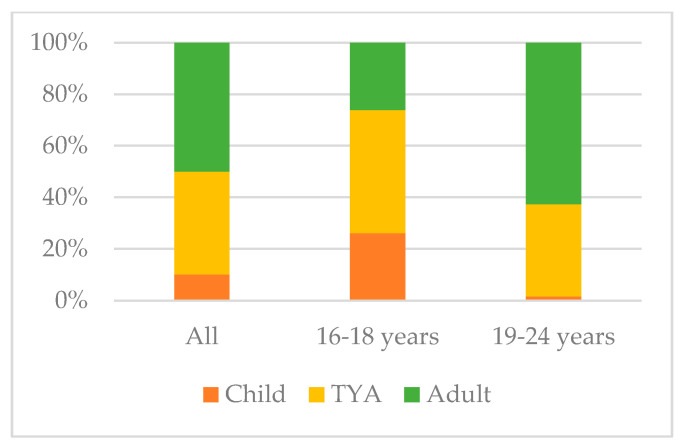
Type of ward most inpatient care delivered in (*n* = 206).

**Healthcare Professionals**
When comparing age cohorts, more young people aged 19–24 years were given a choice in where they received treatment and care (*n* = 82/160, 51%) than those aged 16–18 years (*n* = 27/87, 31%), *p* = 0.009. Young people who were given the choice of where to be treated generally felt they had made the right decision (*n* = 101/109, 93%). Family and partners provided most of the support for making the decision (*n* = 101/147, 69%), whereas healthcare professionals were only reported by 31% (*n* = 46) of young people as helping them make the choice. There was evidence of a difference in where young people were treated depending on their age (*p* < 0.001). Most young people aged 19–24 years received inpatient care in either an adult or specialist TYA unit; however, two were treated in a children’s ward (Figure 1). Just over a third (*n* = 91/244, 36%) of young people reported receiving care in another hospital. Most young people felt it was very/slightly important that areas in the hospital they were treated in were suitable for young people (*n* = 179/247, 72%), and around half (*n* = 118/247, 48%) felt it was very/slightly important to meet or speak to other young people during their treatment. There was no evidence of difference between age or where young people were treated with this perception (*p* > 0.05). See additional details in the Supplementary Materials, Table S1a,b. 
“Having received treatment in both a specialized young adult/teenage unit at the [TYA-PTC name] and then moving my treatment to [local hospital] where I am being treated in an adult unit, I can say that it is definitely preferable to have treatment in the young adult unit. I changed hospitals because of the distance from my home and difficulties getting to the [TYA-PTC name], not because of their standard of care. I felt more comfortable in the teenage unit and I feel the staff were better equipped to deal with young adults and the specific problems we face. Receiving treatment in the adult unit now is not a problem and the staff are very good at making sure my needs are met, but I felt it was better and more comfortable in the teenage unit.” (Female, 21 years, joint care.)Healthcare professionals reported that the place of care depended on the type of tumor, complexity of the cancer, and availability of the TYA-PTC in the region. Sometimes, choice of care was influenced by young people or healthcare professional views, e.g., choosing to stay in a designated hospital due to medical consultant preference. There were differences in opinion on whether to prioritize local treatment or care in an age-appropriate environment. However, sometimes, the availability of a clinical trial defined whether young people were cared for in a TYA-PTC or designated hospital. If a young person was on a clinical trial, joint care was perceived as a challenge because some of the clinical trial activity could only be undertaken at the hospital where the young person was recruited.


“It’s quite reasonably cut and dry in terms of where you’ll be treated depending upon your age.”

Despite the healthcare policy stating clear guidance on place of care by age, some professionals felt that some young people aged 16–18 were still not being referred to the TYA-PTC they remained in the designated hospitals.
Joint care was available for those who chose to have treatment in a TYA-PTC, but the level of joint TYA-PTC and designated hospital input varied depending on individual circumstances. Healthcare professionals in the designated hospitals reported that there was no established joint care model and, in some cases, no joint clinical decision-making, as each hospital had their own site-specific MDTs. Joint care was described by some healthcare teams as tiered: level 1 indicates supportive care only, and level 3 indicates all care after diagnosis. However, no formal definition/classification of tiers existed.

### 3.2. Treatment


**Young People**

**Healthcare Professionals**
A total of 171/243 (70%) young people felt they were involved in the decision on what treatment they were going to have, and 227/246 (92%) found that the explanations of their treatment and preparation for side effects were helpful. Overall, 200/243 (82%) young people rated their treatment teams as excellent or good for helping them prepare for treatment.


“Letters keeping me informed of appointments, arrangements for COVID PCR tests, my main doctor answering all of my questions, giving me options for treatment and making sure I understand everything being said. Being seen by the same doctor for treatment/results who knows my situation.” (Female, 24 years, joint care.)Healthcare professionals reported that the choice of the type of treatment was determined by cancer type and disease severity, and decision-making lay with the MDT.

### 3.3. Healthcare Professional Support


**Young People**

**Figure 2:**
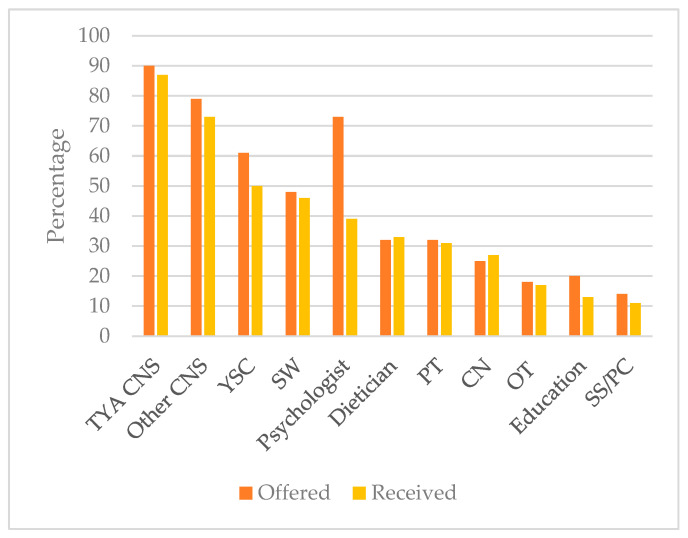
Number of young people being offered and receiving support from members of the MDT. CN: community nurse; CNS: clinical nurse specialist; Education: educational mentor; OT: occupational therapist; PT: physiotherapist; SS/PC: symptom support or palliative care; SW: social worker; TYA: teenage and young adult; YSC: youth support coodinator.

**Figure 3:**
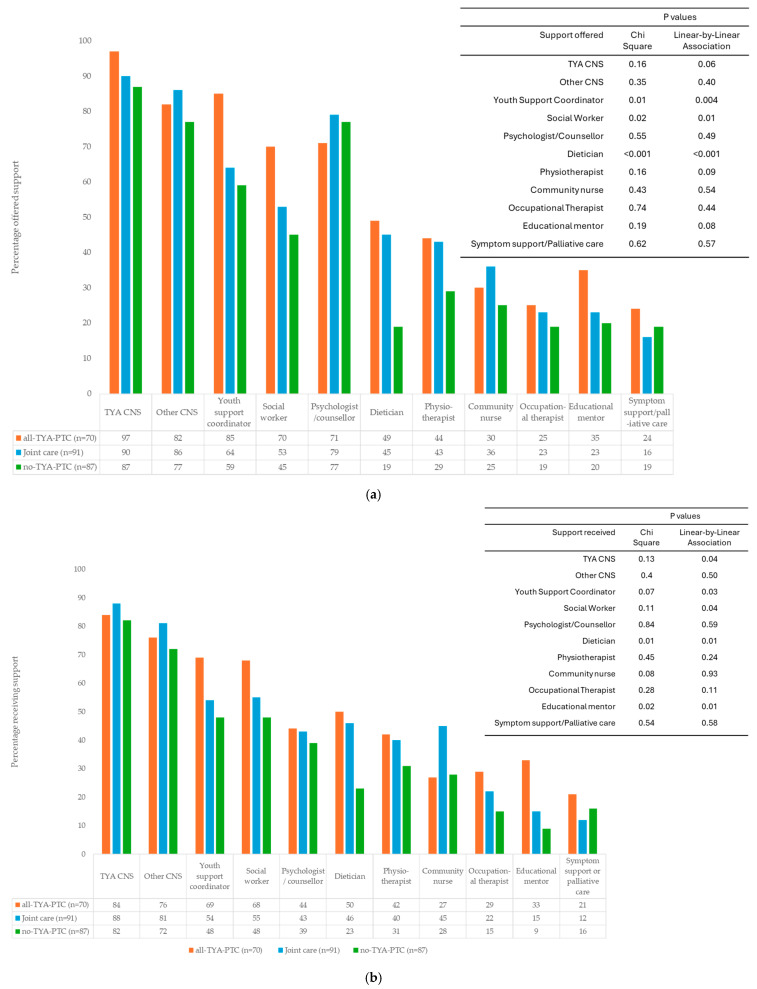
(**a**) Percentage of young people *offered* support from members of the multidisciplinary team according to their place of care. Chi^2^ and linear-by-linear association. (**b**) Percentage of young people *receiving* support from members of the multidisciplinary team according to their place of care. Chi^2^ and linear-by-linear association. CNS: clinical nurse specialist; TYA: teenage and young adult.

**Healthcare Professionals**
The professionals most young people reported being offered and receiving care from were clinical nurse specialists (CNSs), either TYA- or cancer-specific. While 73% (*n* = 171/234) of young people were offered access to a psychologist or counsellor, only 39% (76/196) received support (Figure 2). Figure 3a,b show the proportion of young people being *offered* and *receiving* support from members of the MDT depending on their place of care. There was no statistical evidence of a significant difference in the *offer* of support from a CNS, TYA CNS, psychologist, education mentor, physiotherapist, or community nurse, occupational therapy and symptom support/palliative care or place of care (detailed numbers in the Supplementary Materials, Table S2).

“My clinical Nurse was amazing at acknowledging I was transgender and ensuring I was respected and called the right pronouns by my nurses giving my treatment” (young person, 20 years, joint care).

There was evidence of a significant difference in the *offer* of support from youth support coordinators, social workers and dieticians between categories of care, and the linear-by-linear association test confirmed a significant linear trend, with decreasing levels of support offered (all-TYA-PTC > joint-care > no-TYA-PTC).

“Physio. There was about 20 min of physio post-surgery in hospital and nothing after that.” (Male, 20 years, no-TYA-PTC.)

“If possible—support from a dietician/nutritionist would be beneficial to those who have had a thyroidectomy. I am very confused on how to navigate my medication and what to eat in order to optimise the levothyroxine medication.” (Female, 20 years, joint care.)


There was no statistical evidence of a difference between categories of care for those *receiving* support from the TYA CNS;, however, there was a trend of decreasing support across categories of care (all-TYA-PTC>joint-care>no-TYA-PTC, *p* = 0.04). There was no evidence of a difference in *receiving* support from a CNS, community nurse, occupational therapy, or symptom support, nor was there any evidence of decreasing support across categories of care. Despite evidence of differences in *offers* of support, there was no evidence of a difference in support *received* from social workers (*p* = 0.109), although there were decreasing levels of support across categories of care (all-TYA-PTC>joint-care>no-TYA-PTC, *p* = 0.04). There was a trend of a difference in YSC support between categories of care (0.07) and decreasing support across categories of care (all-TYA-PTC>joint-care>no-TYA-PTC, *p* = 0.03). There was evidence of significant differences in the level of support and support decreasing across all-TYA-PTC>joint-care>no-TYA-PTC for education mentors (*p* = 0.015, linear-by-linear association, *p* = 0.006) and dieticians (*p* = 0.01, linear-by-linear association, *p* = 0.005, Figure 3b, Supplementary Materials Table S2).All places of care aimed at guiding young people effectively through their treatment journey, either through the outreach coordinator, disease-specific clinical nurse specialist or TYA nurse specialist.

Healthcare professionals in the TYA-PTC and designated hospitals reported differences in the amount and type of support they provided. In the designated hospitals, young people had contact with members of the local MDT, the outreach team from the TYA-PTC and, where necessary, mental health practitioners.

In the TYA-PTC, there were specialist nurses, physiotherapists, occupational therapists, youth support coordinators and a TYA MDT coordinator providing oversight of care. Designated hospitals provided essential treatment but not always the comprehensive care that the TYA-PTC did. Physiotherapy was provided at the TYA-PTC, but if long-term rehabilitation was needed, young people were referred to locally, which did not always provide the same specialist care.

### 3.4. Mental Health


**Young People**

**Figure 4:**
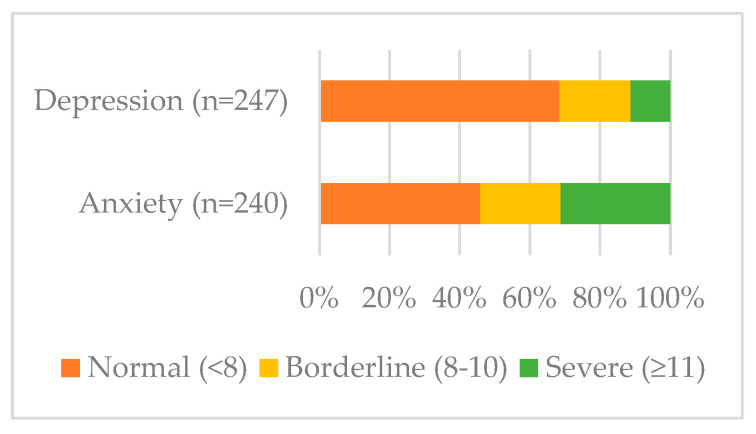
Prevalence and severity of anxiety and depression.

**Healthcare Professionals**
A total of 70/247 (28%) young people reported receiving treatment for mental health problems before their cancer diagnosis. Over half of young people were rated as having borderline or severe anxiety, while a third scored within the range for depression (Figure 4). There was no evidence of a difference according to place of care or age; however, young people who had a pre-existing mental health problem were more at risk of severe anxiety, with just over half of all young people with a pre-existing problem reporting severe anxiety (Supplementary Materials, Table S3a–c
*p* < 0.001).

Figure 2 shows the difference in psychological support offered and received. Psychosocial support was also received via youth support coordinators (*n* = 103/208, 50%) and social workers (*n* = 94/205, 46%).

“I found it difficult to place myself in the category of cancer patient as my cancer was never explicitly confirmed. I feel a bit out of sorts or isolated as a result.” (Female, 23 years, joint care.)Mental health services were available for all young people who were referred to the TYA-PTC. However, TYA-specific psychology services only addressed issues relating to the cancer diagnosis and did not treat pre-existing mental health issues.
In the designated hospitals, young people were supported by outreach nurses, but there were not always services for psychological support. A further challenge was that mental health services were sometimes restricted to children (<16 years) or adults (>18 years). They therefore needed to access psychological support from the local TYA-PTC.

Mental health support was often provided by professionals remotely in the third sector, but there was the perceived risk of potentially missing young people’s needs through telephone or virtual appointments:


“There’s a lot you can pick up by visibly seeing patients face to face and their body language and how they respond to the situation they’re in and how they’re coping emotionally […] But then when you phone then, they often don’t pick up the phone, so it gets to texts and WhatsApp or you find that you’re the one, like chasing when they’re in hospital next and you’ll turn up and try and engage with them in that way.”


All healthcare professionals believed that more mental health support for young people and their family should be available.

### 3.5. Fertility and Sexuality


**Young People**

**Figure 5:**
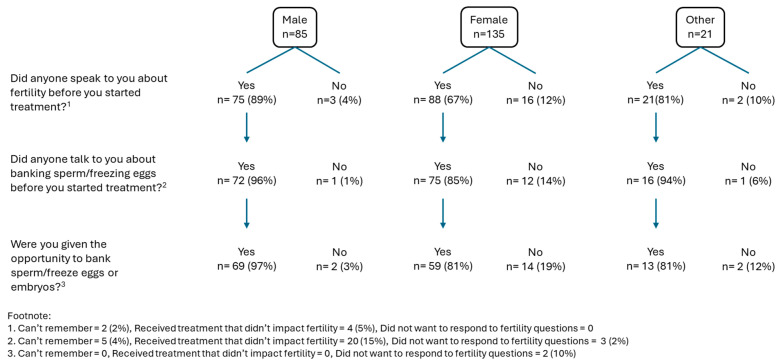
Fertility discussions according to gender.

**Figure 6:**
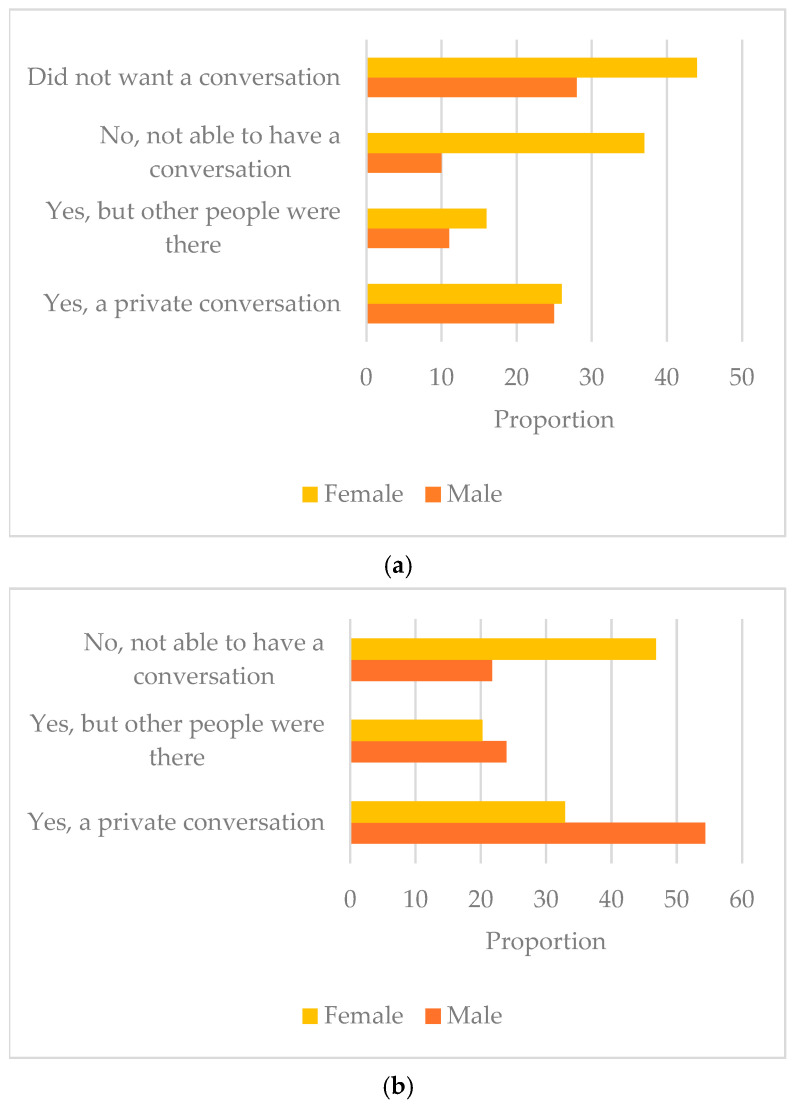
(**a**) Ability to have a conversation about sexual relationships according to gender (*n* = 197). (**b**) Ability to have a conversation about sexual relationships according to gender for those who wanted to have conversation (*n* = 125).

**Healthcare Professionals**
Fertility discussions occurred more in males than females, and while most of those who had all-TYA-PTC care had a fertility discussion, this was not statistcally significantly different by place of care (*n* = 61 (88%) versus 66 (74%) in joint care and 57 (66%) in no-TYA-PTC care, Supplemetary Materials, Table S4a). However, when they had the discussion, the majority were given the opportunity to bank/freeze sperm or embryos (Figure 5). The way the treatment team handled young people’s experience of fertility was rated by 138 young people, of whom 114 (83%) responded it was excellent/good. A total of 88/216 (41%) young people were able to have a conversation with a healthcare professional about the impact of cancer on sexual relationships (overall, *n* = 80, 37% did not want to talk about sex with their treatment team, and this was similar in terms of gender, with females at 36% versus males at 38%, Figure 6a, Supplementary Materials, Table S4b). There was no evidence of a difference according to place of care or age, but more females (*n* = 37, 30%) could not have a conversation in comparison to males (*p* = 0.05, Supplementary Materials, Table S4b). This was even more apparent when looking at gender differences for those who wanted to have a conversation but were unable to, with 47% of females versus 22% of males (Figure 6b). Of 211 who responded, 124 (59%) young people were advised to practice safe sex. There was no evidence of a difference according to where young people were treated or gender differences, but fewer young people aged 16–18 years were advised to practice safe sex: *n* = 32/68, (47%) versus 92/143 (64%) of 19–25-year-olds, (*p* = 0.008, Supplementary Materials, Table S4c). No young people left comments about their experiences of fertility or discussions around sex.
Professionals perceived that there were excellent pathways to ensure young people had access to fertility preservation at the time of their diagnosis and that it was a core topic discussed in all TYA MDT meetings.

Some hospitals had implemented fertility coordinators to ensure access to fertility preservation, which was important when fertility services were not available in the designated hospitals.

There was a perception that it was in young people’s best interests to be in an age-appropriate environment to ensure discussions on sensitive issues were held, for example, on sexual issues and illicit drug use. Children’s wards were not viewed as comfortable environments for these sensitive subjects to be discussed.


### 3.6. Clinical Trials


**Young People**

**Healthcare Professionals**
Forty-seven (19%) young people reported being offered entry into a clinical trial. There was no evidence of a difference according to place of care, but it was slightly higher in those aged 16–18 years (*n* = 22, 25%) in comparison to those aged 19–25 years (*n* = 25, 16%). Of those offered entry into a clinical trial, 34 (72%) consented and took part, two (4%) consented but did not take part, nine (19%) refused to participate and two (4%) could not remember if they had participated. Forty-three (91%) rated the discussion they had about clinical trials as excellent/good. No young people left comments about clinical trials.There was the perception that participation in clinical trials required young people to be in a TYA-PTC. In some regions, there were regular meetings reviewing upcoming clinical trials, so all young people were matched to an appropriate trial when they were newly diagnosed. This was facilitated in some regions where there was a specific TYA research nurse for trial enrolment, which was viewed as increasing clinical trial participation.

Healthcare professionals felt they did a good job offering young people entry into clinical trials, but some believed that those in designated hospitals did not mention clinical trials to young people.

### 3.7. Coordinated Care


**Young People**

**Figure 7:**
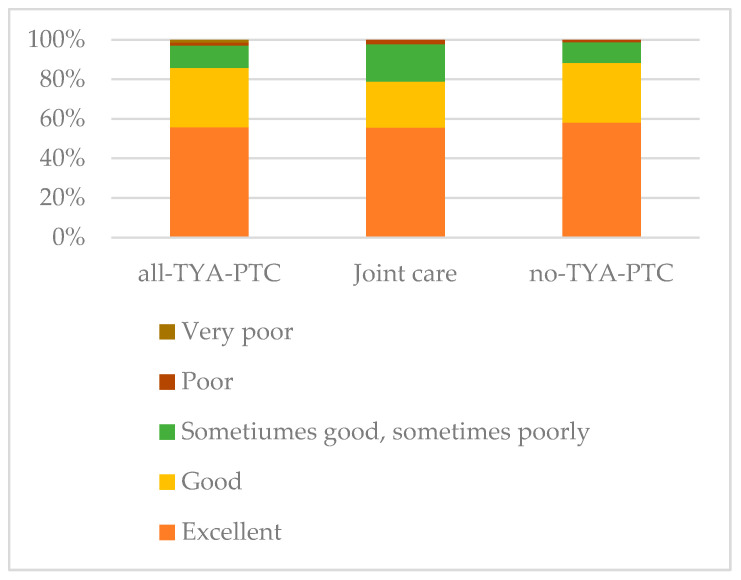
Young people’s assessment of the coordination of care.

**Healthcare Professionals**
Irrespective of where young people were treated, the majority reported that the different professionals involved in their care worked together (Figure 7), and 224 (90%) rated the service they received as excellent or good. However, some of the free-text comments identified aspects of care where coordinated care could be improved. These were all from young people who received joint care.

“Lack of communication between different trusts meant a lot of effort for me, whilst completing a course of radiotherapy. I had to communicate most of what was going on to all of my different healthcare professionals, doing the majority of my own administration.” (Female, 22 years, Joint Care)

“Better communication between specialist and local hospital. More thorough care plans, and being able to access these. More structured care/ care plans and a quicker production of them, particularly for unplanned visits (such as for infections). A higher interest to be given in how I feel about my care and what I want (both a disregard/ ignoring what I want, as well as dismissing me to discuss my care with my mother instead, despite being 18 myself).” (Male, 18 years, Joint Care)

“Communication from the hospital that performed my surgery with my GP, me, and the hospital I was discharged to, and for that information to be checked as there were errors.” (Male, 19 years, Joint Care)Professionals across each network had implemented various mechanisms to improve the coordination of care, for example, appointing outreach nurses to provide a link between the TYA-PTC and designated hospitals. However, this depended on the designated hospitals informing the TYA MDT that there was a young person newly diagnosed with cancer in their hospital so that the outreach nurse could be made aware of them. There was also the perception that while professionals in the designated hospitals communicated with the TYA-PTC, the TYA-PTC was not always good at communicating with the designated hospitals. The passage of information between organizations was often a challenge due to the number of electronic health records in use. Professionals across the network recognised the importance of building relationships between the TYA-PTC and the designated hospitals so that there could be effective joint care.

## 4. Discussion

The current study was part of a wider evaluation of cancer services in England and Wales for teenagers and young adults. Our earlier analysis showed that there was no evidence of significant differences in outcomes according to the type of cancer unit young people were treated in [18], and we surmised that this could potentially be explained by more coordinated care [19]. This aspect of the study focused on young people’s experiences of care and how this compared to healthcare professionals’ perceptions on how care was delivered. A critical finding of this analysis is that there was no evidence of a difference in the proportion of young people receiving support from core components of age-appropriate care such as the TYA CNS and social workers, with just a trend of lower levels of support from youth support coordinators across the three categories of care (in favor of the all-TYA-PTC and joint care groups). Similar rates of support from physiotherapists, psychologists, occupational therapists, and symptom control/palliative care were observed across different categories of care, albeit with less than half of patients receiving support from these professionals. Of interest is the fact that these professional groups do not have substantial third-sector endorsement in the UK like TYA CNSs, youth support coordinators and social workers, where nationally they are mostly funded by the third sector (non-governmental organizations). The similar levels of age-appropriate support across different care categories were most likely because all the cohorts were known to the TYA MDT due to our recruitment method, and this is a critical finding of the study, leveraging the TYA MDT as crucial to care delivery outside of the TYA-PTC [18]. Our results suggest that when young people are known to the TYA-MDT, the TYA-MDT facilitates coordination of age-appropriate outreach services for young people not being treated solely in the PTC, and this potentially explains why there is no evidence of differences in outcomes based on location of care.

Generally, young people rated their experiences of receiving care highly. However, healthcare professionals revealed several difficulties and challenges in delivering TYA care. This is likely to be due to professionals trying to deliver care according to recommendations laid out in the current TYA service specification and knowing when standards of care are not as high as they could be. The expectations of young people may be lower due to their limited experience of accessing health care. Young people rating their care as good possibly reflects the mechanisms that healthcare professionals are implementing across their networks to facilitate high and equitable care wherever young people are treated. What we could not ascertain in this study is what these mechanisms were and how sustainable they were. This was identified as a challenge to care closer to home and coordinated care in an early study evaluating the implementation of TYA services in one region in England [24]. The availability of services, especially for 19–24-year-olds, depended upon the knowledge and relationship that had been developed by the young person’s key workers, especially in primary care. If the key worker was not available, then young people no longer had access to this care [24].

### 4.1. Place of Care

Organizational, clinical and individual factors shaped whether young people were offered a choice of where to receive care. Healthcare professionals felt that referral to TYA-PTCs was inconsistent and driven by cancer type and severity, which was supported to some extent by young people’s responses, with fewer than half reporting that they were given a choice. Very few identified a healthcare professional as helping them reach that decision. Choice of care setting is central to the Improving Outcome Guidance, [5] with ‘unhindered access’ specified for those aged 19–24 years, which was reflected in this age group having the most choice. The current service specification continues this emphasis, adding a requirement to document choice in the TYA MDT referral proforma [22]. Shared decision-making on location of care is recognised as a core component of TYA cancer policy globally [25,26,27].

While more 19–24-year-olds were treated in adult units as expected, some young adults were on children’s wards, which is a concern. Self-reporting limits interpretation, as specialist TYA units developed with young people may appear ‘child-like’ to some. ‘Joint care’, now included in the service specification to enable care closer to home [28] and aligned with the NHS 10-year plan, was discussed by professionals, but survey questions were insufficiently detailed to capture young people’s experiences of it. Open-ended responses indicated that care closer to home was broadly valued, but coordination difficulties within joint care arrangements warrant more in-depth exploration.

### 4.2. Treatment

Young people felt involved in treatment decisions, with only a small proportion wanting greater involvement. Healthcare professionals noted a perception that young people sometimes felt they lacked real choice. Notably, communication of side effects was not mentioned by professionals, yet young people found explanations about side effects helpful, consistent with evidence that young people value greater involvement in supportive care decisions [29]. The complexity of decision-making, involving families, friends and professionals [30], was not fully captured here and warrants further research, including the role of this ‘triad’ in treatment and supportive care choices.

### 4.3. Healthcare Professional Support

While healthcare professionals described multidisciplinary services, discussions tended to focus on clinical rather than holistic care, reflected in survey gaps around allied health input. Only a third of young people were offered dietician or physiotherapy support, with significantly lower rates for those without TYA-PTC access. The service specification recommends 0.8 and 1.0 WTE for dieticians and physiotherapists respectively in TYA-PTCs, yet only 9% of TYA-PTCs met the dietetics standard [31], and a national shortage of physiotherapists [32] means many young people who would benefit from rehabilitation are not receiving it. No staffing requirements exist for these roles in designated hospitals, likely explaining the low rates of offer and receipt. Both physical activity and nutrition can meaningfully improve treatment-related symptoms such as pain and fatigue, and early specialist input has documented long-term benefits [33,34,35,36].

Despite professional perceptions that designated hospitals provide only essential cancer care, young people’s survey responses showed no evidence of a difference in TYA CNS, CNS, or social worker support across care categories. Youth support coordinators and educational mentors, roles unique to TYA-PTCs, were offered to young people across settings, indicating that network-level mechanisms rather than hospital-level provision are driving equitable access. Where no differences in offer or receipt are found, the key question is what barriers exist between offer and uptake: is this a matter of access or of young people’s awareness of the value of available support? In a system under pressure to deliver timely cancer treatment, these roles may seem non-essential, yet youth support coordinators provide a distinct pastoral function [37], and educational mentors support young people to remain in education, facilitating social reintegration when treatment ends [38,39]. Stronger evidence from larger evaluations is needed to justify further investment in these posts.

### 4.4. Mental Health

Nearly a third of young people had pre-existing mental health problems before diagnosis, yet professional perceptions were that available mental health services focused on post-diagnosis symptoms, disregarding prior conditions. Young people with pre-existing conditions had the highest rates of severe anxiety post-diagnosis, indicating they should be prioritized for early psychological support. Fewer young people received psychological support than were offered it, possibly reflecting stigma, long NHS waiting times [40], or pandemic-related redeployment of psychologists. The TYA-PTC service specification recommends 1.0 WTE for psychologists, a requirement not extended to designated hospitals or joint care models [41]. Developing psychological interventions is the top TYA research priority [42], and unmet psychological need is well documented [43]. Given that a lack of support can impair social reintegration after treatment, this area requires much greater attention and systematic evaluation.

### 4.5. Fertility and Sexuality

Fewer than half of young people discussed the impact of cancer on sexual relations with their healthcare provider, and when they did, conversations focused on safe sex. Professionals expressed concern that pediatric professionals would not feel equipped for such discussions, reflected in younger respondents being less likely to receive safe sex advice. No difference by care setting is reassuring for equity but does not indicate adequacy; previous research has highlighted that questions around sexual function (e.g., impact of chemotherapy on sexual experience) were seldom or never addressed by clinical teams [44]. It is unlikely that clinicians in children’s cancer units would have competence in these conversations.

In contrast, most young people reported having a fertility discussion. Although professionals perceived fertility pathways to be accessible, a quarter of young people were not offered the option of banking or freezing. Place of care may further affect access to fertility services, as designated hospitals were noted to provide fewer of these. Fertility has historically been a primary driver of specialist TYA cancer care globally [8,9,25,26,45,46], and while no evidence of difference by care setting was found, the current study indicates that access to fertility preservation remains inconsistent; clinical pathways must ensure that it is offered to every young person who wants it.

### 4.6. Clinical Trials

Only a fifth of young people reported being offered a clinical trial, far below the NHS target of 50% by 2025 [47]. This discrepancy likely reflects limited trial availability rather than a failure to offer, as professionals reported routinely offering trials where available and the young person was eligible. Consistent with international trends [48,49], more 16–18-year-olds were offered trials than 19–24-year-olds, reflecting greater pediatric trial availability and the more diverse cancer spectrum in older young people receiving care across a wider range of settings.

Young people aged 15–24 have historically been underrepresented in clinical trials relative to children and older adults [50,51,52]. Fern et al. identified five criteria needed to improve access: availability (fewer trials exist for young people); accessibility (young people may not be treated at sites where trials are open); awareness (by both young people and professionals); acceptability (professionals’ confidence in the trial and suitability of design); and appropriate age eligibility reflecting the epidemiology of the cancer [48]. Over a decade on, these barriers remain largely unresolved (unpublished NIHR data). The service specification’s advocacy for joint care creates additional regulatory challenges for trial inclusion, though the NHS 10-year plan’s focus on care closer to home may drive the regulatory reform needed [28].

### 4.7. Coordinated Care

A primary aim of BRIGHTLIGHT_2021 was to explore healthcare professionals’ perceptions of care coordination between TYA-PTCs and designated hospitals. Healthcare professionals were striving to provide coordinated care but lacked resources for key mechanisms such as outreach nurses [19]. Young people reported overall satisfaction and perceived their teams as working well together; however, free-text responses revealed instances of poor coordination, predominantly in joint care, that were highlighted as needing improvement. Effective TYA cancer care requires more than adult–pediatric oncology collaboration [3,53,54]; it demands genuinely integrated medical and psychosocial care. Evidence from North America identifies patient navigators and nurse case management as the most commonly employed and effective coordination strategies [55]. More detailed investigation into young people’s experiences of joint care is required to identify scalable models of collaboration.

While this study was a national evaluation of TYA cancer services from the perspective of young people and healthcare professionals, it does have several limitations. Although there were a sizeable number of participants in both studies, this reflected 26% of young people eligible during the recruitment period, and healthcare professionals were predominantly from TYA-PTCs. Further, all participants were known to the TYA-MDT, and so we were unable to capture the experiences and outcomes of young people receiving no TYA oversight on their care at all. While we included a sizeable number of young people, it was not sufficiently powered to show significant differences between categories of care for support by the different healthcare professions; however, the trends are consistent with higher support if young people have contact with a TYA-PTC (all or some). Professionals’ perspectives may be biased toward the specialist care they deliver and miss some of the facilitators and barriers experienced in the designated hospitals. Likewise, we may have only captured the experiences of engaged young people and not those who are traditionally classed as hard-to-reach, e.g., people with English as a second language. Our recruitment method was designed to overcome professional gatekeeping, which affected recruitment in our first BRIGHTLIGHT evaluation [17], and our current method depended on young people receiving and opening the invitation, completing the survey and returning it. Young people who were homeless or had unstable living conditions would potentially not receive it, and those who could not communicate in written English would not be able to complete it. Future studies need to give careful consideration on how recruitment can engage these young people, as their experiences are seldom represented in research. The second limitation of this analysis was that each study focused on a different aspect of care (outcome and experience versus perception of coordination), so it was complex aligning the two datasets during integration. While both datasets offered valuable insights, their differences in detail and scope had the potential to not fully capture an experience or perspective. The final limitation was the exclusion of Scotland in the integration. We were able to recruit young people to the survey but were unable to conduct the qualitative study, so we had no information to provide context for young people’s experiences of care. Ensuring inclusion of all the devolved nations in the UK will be central to research in the future, and strategies to harmonize ethical and regulatory approval systems are required to manage the resource implications of opening across devolved nations. Despite these limitations, we have provided a comprehensive understanding of the delivery of cancer care from across England and Wales, identifying where services supported young people well but also where improvements could be made.

## 5. Conclusions

The comparison between young people’s experiences of care and healthcare professionals’ perceptions of the delivery of care are overall discordant. Generally, young people reported being satisfied with the care they received, irrespective of it being in a specialist TYA unit or care closer to home, and this is most likely due to similarities in the provision of age-appropriate specialists such as TYA-CNS and youth support coordinators. Despite location of care, some, if not all, aspects of age-appropriate care have been received by the majority of the BRIGHLIGHT_2021 cohort, reflecting nationwide professional commitment and charitable investment in TYA cancer care. This suggests that young people’s positive experiences are sustained by networked mechanisms such as TYA-MDT involvement, outreach links, and access to professionals in age-appropriate roles (e.g., TYA-CNS, youth support coordinators). Nevertheless, gaps persist in communication and coordination, particularly within joint care models, and in the involvement of allied health professionals such as dieticians and physiotherapists, whose input is essential for rehabilitation and return to normal life. Strengthening these areas will require continued investment in workforce capacity and digital infrastructure to support genuinely coordinated, developmentally appropriate TYA cancer care.

## Figures and Tables

**Table 1:** Young people’s characteristics (*n* = 250).

Characteristic		*n*	Number (%) (Unless Specified Otherwise)
Age at diagnosis (years)	Mean years (SD)	250	19.7 (2.7)
	Median (range)		20 (16–24)
Age group	16–18 years	250	89 (36)
	19–24 years		161 (64)
Gender	Male	250	89 (36)
	Female		140 (56)
	Other		21 (8)
Ethnicity	White	250	207 (83)
	Other		43 (17)
Socioeconomic status (IMD fifth)	1—most deprived	224 *	65 (26)
	2		30 (12)
	3		75 (30)
	4		22 (9)
	5—least deprived		32 (13)
	Missing		26 (10)
Marital status	Married/civil partnership	248	<5 (<1)
	Cohabiting		21 (8)
	Single/divorced		225 (90)
	Missing		2 (<1)
Type of cancer	Bone and soft tissue sarcoma	250	20 (8)
	Carcinomas (not skin)		46 (18)
	CNS		15 (6)
	Germ cell		35 (14)
	Leukemia		22 (9)
	Lymphoma		92 (37)
	Melanoma		11 (4)
	Other		9 (4)
Prognostic score **	>80%	244	191 (76)
	50–80%		49 (20)
	<50%		4 (2)
	Missing		6 (2)
Location	East Midlands	249	18 (7)
	East of England		29 (12)
	London		55 (22)
	Merseyside		17 (7)
	Northeast		15 (6)
	Northwest		20 (8)
	Southwest		24 (10)
	Thames Valley		16 (6)
	Wessex		8 (3)
	West Midlands		10 (4)
	Yorkshire		29 (12)
	Wales		9 (4)
Long-term condition prior to cancer	Yes	249	44 (18)
	No		205 (82)
	Missing		1 (<1)
Category of care	all-TYA-PTC	248	70 (28)
	Joint care		91 (37)
	no-TYA-PTC		87 (35)

IMD: indices of multiple deprivation; CNS: central nervous system. * Calculated from home postcode in England at the time of diagnosis, and not all participants were resident in England. ** Prognostic score based on anticipated 5-year survival.

## Data Availability

Data that is not held under license with NHS England will be available from late 2025 when the primary analysis is complete. We welcome collaboration; for general data sharing enquiries, please contact RMT (rtaylor13@nhs.net).

## Supplementary Materials

The following supporting information can be downloaded at: https://www.mdpi.com/article/10.3390/curroncol33040211/s1, Table S1a: Age differences according to the place of care (*n*, %); Table S1b: Importance of being with peers according to category of care (*n*, %); Table S2: Number (%) of young people offered and receiving support from members of the multidisciplinary team according to place of care (numbers do not always total 250 due to missing data); Table S3a: Prevalence and severity of anxiety and depression according to category of care (*n*, %); Table S3b: Prevalence and severity of anxiety and depression according to age (*n*, %); Table S3c: Prevalence and severity of anxiety and depression according to history of mental health or emotional wellbeing problems before diagnosis; Table S4a: Conversations about fertility and sexuality according to category of care (*n*, %); Table S4b: Difference in the ability of have conversations about sexual relations according to gender (*n*, %); Table S4c: Group differences in advice to practice safe sex (*n*, %); Section S1: Unabridged discussion.
