# Supplementary material for: Mixed-Methods Evaluation of the Delivery of Cancer Care to Teenagers and Young Adults in England and Wales: BRIGHTLIGHT_2021
Source: Curr Oncol. 2026 Apr 10;33(4):211. doi: 10.3390/curroncol33040211 (PMC13114648; doi:10.3390/curroncol33040211)
Supplement: Supplementary file 1 [file curroncol-33-00211-s001.zip › curroncol-4164134-supplementary.pdf]

Supplemental file

Table S1a. Age differences according to the place of care (*n*, %).

		16-18 years	19-25 years	P value
Given a choice where to receive care (n=247)	Yes	27 (31)	82 (51)	0.009
	No	47 (54)	62 (39)	
	Can't remember	13 (15)	16 (10)	
Category of care (n=248)	all-TYA-PTC	22 (25)	48 (30)	.078
	Joint Care	40 (46)	51 (32)	
	no-TYA-PTC	25 (29)	62 (38)	
Type of ward most inpatient care (n=206)*	Children's unit	17 (25)	2 (1)	<0.001
	TYA unit	31 (46)	48 (35)	
	Adult unit	17 (25)	77 (56)	
	Don't know	3 (4)	11 (8)	
Importance of being treated in the same area as other young people (n=247)	Very important	45 (51)	62 (39)	0.315
	Slightly important	24 (27)	48 (30)	
	Less important	10 (11)	32 (20)	
	Not important	7 (8)	14 (9)	
	Don't know	2 (2)	3 (2)	
Importance of speaking to other young people (n=246)	Very important	12 (14)	39 (25)	0.363
	Slightly important	25 (29)	42 (26)	
	Less important	27 (31)	43 (27)	
	Not important	20 (23)	32 (20)	
	Don't know	3 (3)	3 (2)	

*Not all young people received inpatient care

Table S1b. Importance of being with peers according to category of care (*n*, %).

		all-TYA-PTC	Joint Care	no-TYA-PTC	P value
Importance of being treated in the same area as other young people (n=245)	Very important	39 (56)	38 (43)	29 (33)	0.122
	Slightly important	18 (26)	28 (32)	26 (30)	
	Less important	7 (10	16 (18)	18 (21)	
	Not important	4 (6)	5 (6)	12 (14)	
	Don't know	2 (3)	1 (1)	2 (2)	
Importance of speaking to other young people (n=244)	Very important	19 (27)	21 (24)	11 (13)	0.360
	Slightly important	18 (26)	23 (26)	25 (29)	
	Less important	18 (26)	22 (25)	30 (35)	
	Not important	12 (17)	21 (24)	18 (21)	
	Don't know	3 (4)	1 (1)	2 (2)	

Table S2. Number (%) of young people offered and receiving support from members of the multidisciplinary team according to place of care (numbers do not always total 250 due to missing data).

	Offered			$\chi 2$ Linear-by Linear association	Received			$\chi 2$ Linear-by Linear association
	all-TYA-PTC n=70	Joint care n=91	no-TYA-PTC n=87		all-TYA-PTC n=70	Joint care n=91	no-TYA-PTC n=87	
TYA CNS	58 (97)	72 (90)	69 (87)	0.158<br>0.064	58 (94)	71 (88)	64 (82)	0.126<br>0.042
Other CNS	45 (82)	67 (86)	56 (77)	0.347<br>0.4	41 (76)	60 (81)	53 (72)	0.4<br>0.498
Youth Support Coordinator	45 (85)	48 (64)	41 (59)	0.007<br>0.004	37 (69)	36 (54)	29 (48)	0.069<br>0.025
Social worker	38 (70)	39 (53)	32 (45)	0.018<br>0.006	30 (68)	36 (55)	28 (48)	0.020<br>0.109
Psychologist/counsellor	42 (71)	64 (79)	63 (77)	0.553<br>0.488	22 (44)	28 (43)	25 (39)	0.844<br>0.586
Dietician	28 (49)	33 (45)	12 (19)	<.001<br><.001	24 (50)	28 (46)	13 (23)	0.009<br>0.005
Physiotherapist	21 (44)	31 (43)	18 (29)	0.16<br>0.088	19 (42)	26 (40)	17 (31)	0.445<br>0.235
Community nurse	15 (30)	25 (36)	16 (25)	0.433<br>0.543	12 (27)	27 (45)	15 (28)	0.08<br>0.929
Occupational therapist	13 (25)	16 (23)	12 (19)	0.735<br>0.44	12 (29)	12 (22)	8 (15)	0.28<br>0.112
Educational mentor	15 (35)	15 (23)	13 (20)	0.018<br>0.006	11 (33)	7 (14)	5 (9)	0.015<br>0.006
Symptom support or palliative care	10 (24)	10 (16)	11 (19)	0.616<br>0.569	8 (21)	6 (12)	8 (16)	0.54<br>0.575

Table S3a. Prevalence and severity of anxiety and depression according to category of care (*n*, %).

		all-TYA-PTC	Joint care	no-TYA-PTC	P value
	N	66	89	83	
HADS anxiety	Normal	35 (53)	41 (46)	34 (41)	0.532
(n=238)	Borderline	11 (17)	20 (23)	23 (28)	
	Severe	20 (30)	28 (32)	26 (31)	
	N	69	91	85	
HADS depression	Normal	41 (59)	66 (73)	61 (72)	0.283
(n=245)	Borderline	19 (28)	17 (19)	13 (15)	
	Severe	9 (13)	8 (9)	11 (13)	

HADS: Hospital Anxiety and Depression Scale

Table S3b. Prevalence and severity of anxiety and depression according to age (*n*, %).

		16-18 years	19-24 years	P value
	N	86	154	
HADS anxiety	Normal	38 (44)	72 (47)	0.439
(n=238)	Borderline	17 (20)	38 (25)	
	Severe	31 (36)	44 (29)	
	N	89	158	
HADS depression	Normal	57 (64)	112 (71)	0.409
(n=245)	Borderline	19 (21)	31 (20)	
	Severe	13 (15)	15 (10)	

HADS: Hospital Anxiety and Depression Scale

Table S3c. Prevalence and severity of anxiety and depression according to history of mental health or emotional wellbeing problems before diagnosis.

		Yes	No	Possible*	P value
	N	69	143	25	
HADS anxiety	Normal	16 (23)	87 (61)	6 (24)	<0.001
(n=237)	Borderline	18 (26)	24 (17)	11 (44)	
	Severe	35 (51)	32 (22)	8 (32)	
	N	69	149	26	
HADS depression	Normal	39 (57)	109 (73)	19 (73)	0.150
(n=244)	Borderline	18 (26)	26 (17)	5 (19)	
	Severe	12 (17)	14 (9)	2 (8)	

*I think I have emotional/mental health problems but have never sought help

Table S4a. Conversations about fertility and sexuality according to category of care (*n*, %).

		all-TYA-PTC	Joint care	no-TYA-PTC	P value
Conversation before treatment on fertility	N	69	89	86	
	Yes	61 (88)	66 (74)	57 (66)	0.087
	No	3 (4)	5 (6)	11 (13)	
	No impact*	2 (3)	13 (15)	13 (15)	
	Other**	3 (4)	5 (6)	5 (6)	
Conversation about the impact on sexual relations	N	60	76	78	
	Yes, in private	18 (30)	19 (25)	20 (26)	0.135
	Yes, but other people were there	8 (13)	11 (15)	12 (15)	
	No, not able	10 (17)	25 (33)	12 (15)	
	I didn't want to	24 (40)	21 (28)	34 (44)	

*receiving treatment that will not impact fertility; **can't remember or does not wish to answer

Table S4b. Difference in the ability of have conversations about sexual relations according to gender (*n*, %).

		Male	Female	Other	P value
Conversation about the impact on sexual relations	N	74	123	19	
	Yes, in private	25 (34)	26 (21)	6 (32)	0.054
	Yes, but other people were there	11 (15)	16 (13)	4 (21)	
	No, not able	10 (14)	37 (30)	1 (5)	
	I didn't want to	28 (38)	44 (36)	8 (42)	
Advised to practice safe sex	N	74	120	17	
	Yes	47 (64)	69 (58)	8 (47)	0.581
	No	17 (23)	27 (23)	6 (35)	
	Can't remember	10 (14)	24 (20)	3 (18)	

Table S4c. Group differences on advice to practice safe sex (*n*, %).

		n	Yes, advised to practice safe sex	P value
Category of care	all-TYA-PTC	60	39 (65)	0.252
	Joint Care	72	40 (56)	
	no-TYA-PTC	77	43 (56)	
Gender	Male	74	47 (64)	0.581
	Female	120	69 (58)	
	Other	17	8 (47)	
Age	16-18 years	68	32 (47)	0.008
	19-24 years	143	92 (64)	

S1. Unabridged Discussion

The following is the unabridged discussion.

S1.1. Place of care

Organisational, clinical and individual factors impacted whether a young person was offered a choice of where to receive care. Healthcare professionals felt that young people were not being referred to the TYA-PTC, and the decision on where they were treated was based on the type and severity of cancer. This was somewhat supported by young people's responses, with less than half reporting they were given the choice. Furthermore, when young people were asked who helped them with this decision, very few responded that it was a healthcare professional who helped them make the choice of where to be treated. Choice of where to be treated was central to the Improving Outcome Guidance that underpinned the configuration of TYA services in England between 2005 and 2023.[1] Having 'unhindered access' was specified as a requirement for those aged 19-24 years, which was reflected in the current study, where this age group had the most choice. The importance of choice continues in the latest service specification, with the additional directive to document this in the TYA MDT referral proforma.[2] Giving young people a choice and shared decision making on where care is received is an important component of TYA cancer care and included in most of the global policies on TYA cancer.[3-5]

As expected, more 19-24 year olds were treated in adult units than those aged 16-18 years but it was concerning that there were young adults being treated on children's wards. Where young people were treated was self-reported in the survey, so we do not know if this was a children's ward or one of the specialist TYA units. Each Teenage Cancer Trust funded unit in the UK was co-developed with young people so it has age-appropriate décor,[6] which may be perceived as 'child-like' to the older young people in the cohort or someone who has not accessed healthcare previously. It is beyond the scope of this study to discuss the design of specialist TYA units, and what design features make a difference, but young people have reported previously how these units help them through the treatment.[6-8]

'Joint care' has been included in the latest service specification as something that should be offered to young people to enable them to receive care closer to home. This also aligns with the Department of Health and Social Care's recently launched 10 year plan, 'more care will be available on people's doorsteps and in their homes.'[9] 'Joint care' was a concept that was discussed by healthcare professionals but the questions in the survey were too superficial to draw any conclusions on whether young people felt this was available and/or worked. Some of the comments in the open-ended responses suggested that being able to be cared for closer to home was something young people supported but there were aspects of care and the coordination of care that sullied the experience. As the negative comments about coordinated care were predominantly from those receiving joint care, this is something that warrants more in-depth exploration.

S1.2. Treatment

While clinical factors defined treatment options, young people felt involved in the decision-making process. Only a small proportion would have liked to be more involved in their treatment choice. There is a body of evidence exploring young people's involvement in treatment decision making, which shows their preferences vary depending on the type of decision. There was sometimes the perception that they did not have a choice,[10] a view expressed by healthcare professionals in the current study.

Communication of side effects and help in preparing for side effects was not mentioned by healthcare professionals. However, young people reported the explanations about their treatment side effects as being helpful. This perception supports previous findings that young people wanted to be involved more in supportive care decisions.[11] The current study was not designed to capture the complexity of decision-making and communication with healthcare professionals, but it is known to involve families, friends, partners as well as professionals.[12] Further research is needed to understand the role of this 'triad' in aspects of decision-making around treatment.

S1.3. Healthcare professional support

Although services were described by healthcare professionals to be multidisciplinary, there was a tendency for the discussion of services to focus on clinical care rather than holistic (e.g., care by therapists, activity coordinators, social workers and educational mentors). This was reflected in young people's responses in the survey where there were large gaps and variability in the support offered and received by healthcare professions outside of medicine. Only a third were offered care with a dietitian and physiotherapist and this was significantly less for young people who had no access to the TYA-PTC. The Service Specification for the TYA-PTCs in England recommends staffing levels for these professions as 0.8 and one whole time equivalent respectively. However, a survey of dietetic services in TYA-PTCs in England indicated only 9% had this level of resource.[13] There is no national record of the number of physiotherapists working in TYA cancer or treating common TYA cancers which require rehabilitation after surgery, e.g., primary bone tumours, but there is a national shortage of physiotherapists[14] suggesting many young people who would benefit from physiotherapy are not being offered it. There is no recommendation in the Service Specification for Designated Hospitals on staffing levels other than medics and nurses, which possibly explains why only a third were offered support from physiotherapists and dieticians. Physical activity and nutrition help people with cancer overcome some of the symptoms experienced because of treatment, such as pain and fatigue. Having appropriately trained professionals in post who can introduce this to young people early after diagnosis has the long-term benefit potential.[15-18]

Healthcare professionals' perception that designated hospitals provide only essential cancer care and not comprehensive TYA care was not reflected in young people's survey responses. There was no evidence of a difference in support received by the TYA CNS, CNS and social workers across categories of care. Youth support coordinators and educational mentors are roles that are unique to TYA-PTCs. The offer of youth support coordinators was significantly lower for young people in the no-TYA-PTC group; however, when looking at those who received support, there was only slight evidence of a difference across categories of care and a significant trend favouring the TYA-PTC and joint care groups. The offer of educational mentor was similar across groups but those receiving all-TYA-PTC were more likely to have received the support. Support from these TYA specific roles were offered to 16-75% of young people, the fact that young people outside the TYA-PTC were offered and received this support indicates there were mechanisms to ensure equitable provision of services across a network and not just at hospital level. Where we see no differences in the *offer* of support and support *received* the question remains as to the barriers between offer and receipt. Is this lack of access or lack of awareness in young people in the value of the support and not taking up the offer. In clinical services where there is extreme pressure to deliver timely cancer treatment, provision of these roles may not seem essential however, youth support coordinators play an important pastoral service, provided by people with distinct youth skills,[19] and educational mentors ensure young people can remain in education, which can enable social reintegration when treatment ends.[20,21] There is some evidence showing the value of these roles, but more extensive evaluations would provide the justification for more of these posts to be funded.

S1.4. Mental health

Nearly a third of young people reported having pre-existing mental health problems before diagnosis. However, professionals were challenged in that mental health services available to TYA after diagnosis focused on symptoms after the cancer diagnosis, disregarding any pre-existing mental health problems. Young people with a pre-existing mental health condition were found to have the highest rate of severe anxiety after diagnosis, which suggests pre-existing mental health problems should be considered a trigger for early post-diagnosis psychological support.

Fewer young people received care from a psychologist or counsellor than were offered, which may be due to the stigma that still exists around mental health. It was also not possible to know if a young person had accepted the offer but were still waiting for an appointment. This could be a possible explanation for the three quarters of young people who had no access to a TYA-PTC who were offered psychological support of whom, only a quarter received it. This could reflect adult mental health

services in the UK, which are over stretched with long waiting times for assessment.[22] It is also worth noting that these patients were recruited during the pandemic and many psychologists were redeployed to front line COVID services and supporting staff.

The service specification for TYA-PTCs recommends units to provide one whole time equivalent psychologist,[23] which is not a requirement in designated hospitals and is not specified as being essential in the joint care model.[23] Developing psychological interventions is the top research priority for TYA cancer,[24] but what we do not know is how much unmet need there is for this aspect of care, i.e., our results showed the number of young people who were offered and received support, not those who wanted it but were not offered. The young people in our study were 6-months post-diagnosis, so they may not have realised or recognized that they need support. Psychological and emotional support has previously been shown to be an unmet need by young people,[25] and not receiving support can impact young people's ability to reintegrate when treatment ends. It is therefore an aspect of care that requires greater consideration.

S1.5. Fertility and sexuality

Less than half of young people had a conversation about the impact of cancer on sexual relations with their healthcare provider, and these discussions focused on practicing safe sex. One of the concerns healthcare professionals expressed was staff in children's wards would not feel equipped or have experience in talking about sex. This was somewhat supported by young people's responses, where those aged 16-18 years were less likely to be advised about safe sex.

Young people have previously reported fertility and sexuality as being important issues that they want to have conversations with healthcare professionals about[26] but often this was focused on fertility only and not wider sexual health concerns. No evidence of a difference in young people's experiences in these areas according to place of care is reassuring that care is equitable. However, this does not necessarily indicate that the care being delivered reflects young people's needs in this area. Our earlier work exploring sexuality and relationship issues that young people have during and after treatment identified questions that were seldom or never addressed by healthcare professions, for example, "will my chemotherapy affect my ability to orgasm?"[27] As noted by healthcare professionals, it is unlikely that the clinical teams in children's cancer units would have competence in having conversations such as these.

In contrast to discussions on sexuality, most young people reported someone talked to them about fertility i.e., banking sperm or freezing eggs. Although there was the perception among healthcare professionals of fertility services being accessible and pathways being straight forward, a quarter of young people were not given the option to freeze eggs/bank sperm. While there was no evidence of differences regarding having a discussion on fertility according to place of care, place of care could impact the ability to then bank sperm/freeze eggs, as healthcare professionals noted the designated hospitals provided fewer fertility services. Among the ones that did have a conversation about fertility, patients seemed quite satisfied with the fertility treatment service.

Issues around fertility have been one of the key drivers to the development of specialist TYA cancer care globally. This is reflected as it being included as a core component of specialist care in every TYA cancer policy where they exist.[3-5,28-30]However, this needs to go beyond informing young people their fertility will be impacted, to extend to offering sperm banking/freezing eggs. The current study indicates that this does not happen for every young person, and therefore clinical pathways need to ensure it is an option for every young person who wants it.

S1.6. Clinical trials

Healthcare professional's perception was that young people were being offered entry to clinical trials. Despite this, a fifth of young people reported being offered a trial, a number far short of the current NHS target of 50% of TYA being on trials by 2025.[31] The discrepancy in young person data and professional experiences is probably related to trial availability for this age group, and professional experiences are based on always offering young people trial entry where trials are available, and the young person is eligible. Healthcare professional's felt trial entry was mostly offered in the TYA-PTC, but there was no evidence of a difference according to young people's experiences of care. In keeping

with international trends,[32,33] more 16-18 year olds were offered trial options compared to 19-24 year olds, and probably reflects increased availability of trials for children and a higher proportion of 16-18 year olds receiving care in a children's environment. Whereas the older cohort are presenting with a more disparate spectrum of cancer types and receiving care in more locations.

The other key driver to specialist TYA cancer care has been increasing entry into clinical trials. The early pioneers in this area identified that fewer young people aged 15-24 years were enrolled onto a clinical trial in comparison to children and older adults.[34-36] Strategies have been implemented in several countries to increase access to trials which has included, increasing collaboration between adult and paediatric trialists,[32,37] system level changes and toolkits to support the development of more inclusive trials.[5] While some of these initiatives have resulted in improvement in trial entry, this has not been sustained over time. Despite this, TYA remain underrepresented in clinical trials, underpinned by a lack of trial availability and limited industry investment in rare cancers.

In the UK, a target was set to recruit 50% of newly diagnosed young people to research by 2025.[38] As Fern et al. noted in 2014, five criteria needed to be addressed if we were going to improve access to clinical trials: Availability of trials (fewer trials are available for young people); Accessibility of trials (young people may not be treated where relevant trials are open); Awareness (young people and healthcare professionals may not know what trials are available); Acceptability (healthcare professionals have belief in the trial and the trial design is satisfactory for young people); and Appropriate (the age-eligibility for inclusion reflects the epidemiology of the cancer). Over a decade later, many of these criteria have still not been overcome and entry into clinical trials remains lower than in children and older adults (unpublished data from the NIHR). The additional challenge in the UK relates to the Service Specification advocating for Joint Care. The regulations for clinical trials currently cannot easily accommodate this; however, these challenges must be overcome beyond TYA cancer. With the new NHS 10-year plan recommending care closer to home, changes to regulations may come quicker.[9]

S1.7. Coordinated care

One of the primary aims of BRIGHTLIGHT_2021 was to explore healthcare professionals' perceptions of the coordination of care between the TYA-PTC and designated hospitals. We found that healthcare professionals are striving to provide coordinated care but some of the mechanisms required to achieve this, such as outreach nurses, require investment that is not currently available.[39] From the young person's perspective, their survey responses indicated satisfaction with their care and their healthcare team worked well together. However, the free text comments indicated there were instances where care was not coordinated, and this was highlighted as something that needed improvement. It has been recognised that a key feature of a model of TYA cancer care is collaboration between adult and paediatric oncologists,[40-42] but this is too simplistic to address the complex integrated medical and psychosocial care young people require to be able to achieve the same milestones as their peers without cancer. A review of strategies employed in North America to facilitate coordinated care found patient navigators and nurse case management were most commonly employed and this had a beneficial impact on outcomes.[43] More detailed investigation into young people's experience of joint care and how care is coordinated between centres would illuminate further on how effective collaboration can be achieved.

References

[1] National Institute for Health and Care Excellence. *Guidance on Cancer Services: Improving Outcomes in Children and Young People with Cancer.*; NICE, London: https://www.nice.org.uk/guidance/csg7/resources/improving-outcomes-in-children-and-young-people-with-cancer-update-773378893 [Accessed 09/09/16], 2005.

[2] NHS England. *Teenage and Young Adult Cancer Clinical Network Specification.*; NHS England: London, 2023.

[3] *New Zealand Adolescent and Young Adult Cancer Action Plan 2020-2025.*; AYA Cancer Network Aotearoa: Aukland, New Zealand, 2020.

[4] The Canadian Framework for the Care and Support of Adolescents and Young Adults with Cancer**2019**.

[5] Victorian Comprehensive Cancer Centre. Establishing a Cancer Clinical Trial with Age Eligibility Encompassing Adolescents and Young Adults (AYA): AYA Cancer Clinical Trials Research Ethics and Governance Guidelines. **2020**.

[6] The Futures Company Report for Teenage Cancer Trust. *Exploring the Impact of the Built Environment.*; The Futures Company;: https://www.teenagecancertrust.org/sites/default/files/Impact-of-the-Built-Environment.pdf [Accessed 09/09/16], 2010.

[7] Marshall, S.; Grinyer, A.; Limmer, M. The 'Lost Tribe' Reconsidered: Teenagers and Young Adults Treated for Cancer in Adult Settings in the UK. European Journal of Oncology Nursing **2018**, *33*, 85–90.

[8] Kelly, D.; Pearce, S.; Mulhall, A. 'Being in the Same Boat': Ethnographic Insights into an Adolescent Cancer Unit. Int. J. Nurs. Stud. **2004**, *41*, 847–857.

[9] UK Government. Fit for the Future: 10 Year Health Plan for England.

[10] Pyke-Grimm, K.A.; Franck, L.S.; Patterson Kelly, K.; Halpern-Felsher, B.; Goldsby, R.E.; Kleiman, A.; Rehm, R.S. Treatment Decision-Making Involvement in Adolescents and Young Adults with Cancer. Oncology Nursing Forum **2019**, *46*, E22–E37.

[11] Darabos, K.; Berger, A.J.; Barakat, L.P.; Schwartz, L.A. Cancer-Related Decision-Making among Adolescents, Young Adults, Caregivers, and Oncology Providers. Qualitative health research **2021**, *31*, 2355–2363.

[12] Critoph, D.J.; Cable, M.; Farmer, J.; Hatcher, H.M.; Kuhn, I.; Taylor, R.M.; Smith, L.A.M. Is there Scope to do Better? Clinical Communication with Adolescents and Young Adults with cancer—A Scoping Review. Psycho-oncology (Chichester, England) **2024**, *33*, e6317–n/a.

[13] Henry, L.; Aldiss, S.; Gibson, F.; Pugh, G.; Stevens, M. Nutritional Assessment and Dietetic Resource for Children and Young People with Cancer in the United Kingdom. Pediatric blood & cancer **2022**, *69*, e29743–n/a.

[14] The Observer. Shortage of NHS Physio Roles Leaves Patients in Pain as Waiting Lists Soar. The Observer **2024**.

[15] Ito, N.; Petrella, A.; Sabiston, C.; Fisher, A.; Pugh, G. A Systematic Review and Narrative Synthesis of Exercise Interventions to Manage Fatigue among Children, Adolescents, and Young Adults with Cancer. Journal of adolescent and young adult oncology **2021**, *10*, 361–378.

[16] Vasilopoulou, M.; Asimakopoulou, Z.; Velissari, J.; Vicha, A.; Rizogianni, M.; Pusa, S.; Stöven, S.; Ficarra, S.; Bianco, A; Jiménez-Pavón, D. *et al.* Interventions about Physical Activity and Diet and their

Impact on Adolescent and Young Adult Cancer Survivors: A Prisma Systematic Review. Support Care Cancer **2024**, *32*, 342.

[17] Munsie, C.; Ebert, J.; Joske, D.; Ackland, T. The Benefit of Physical Activity in Adolescent and Young Adult Cancer Patients during and After Treatment: A Systematic Review. Journal of adolescent and young adult oncology **2019**, *8*, 512–524.

[18] Skiba, M.B.; McElfresh, J.J.; Howe, C.L.; Crane, T.E.; Kopp, L.M.; Jacobs, E.T.; Thomson, C.A. Dietary Interventions for Adult Survivors of Adolescent and Young Adult Cancers: A Systematic Review and Narrative Synthesis. Journal of adolescent and young adult oncology **2020**, *9*, 315–327.

[19] Cable, M.; Soanes, L.; Whelan, M. Determining Domains of Practice for Youth Support Co-Coordinator Work in Teenage/Young Adult Cancer Care in United Kingdom. Journal of adolescent and young adult oncology **2023**, *12*, 758–764.

[20] Pini, S.; Gardner, P.; Hugh-Jones, S. How Teenagers Continue School After a Diagnosis of Cancer: Experiences of Young People and Recommendations for Practice. Future oncology (London, England) **2016**, *12*, 2785–2800.

[21] Mattock, R.; Martin, A.; Beckett, A.E.; Lindner, O.C.; Stark, D.; Taylor, R.M. Impact of a Cancer Diagnosis on Educational, Employment, Health-Related Quality of Life, and Social Outcomes among Young Adults: A Matched Cohort Study of 401 Cancer Survivors Aged 15–24 in England. Social science & medicine (1982) **2025**, *376*, 118078.

[22] Fernando, A.; Tokell, M.; Ishak, Y.; Love, J.; Klammer, M.; Koh, M. Mental Health Needs in Cancer – a Call for Change. Future healthcare journal **2023**, *10*, 112–116.

[23] NHS England. *Specialist Cancer Services for Children and Young People: Teenage and Young Adult Designated Hospitals*.; NHS England: London, 2023.

[24] Aldiss, S.; Fern, L.A.; Philips, R.S.; Callaghan, A.; Dyker, K.; Gravestock, H.; Groszman, M.; Hamrang, L.; Hough, R.; McGeachy, D. *et al.* Research Priorities for Young People with Cancer: A UK Priority Setting Partnership with the James Lind Alliance . BMJ Open **2019**, *9*, e028119.

[25] Tsangaris, E.; Johnson, J.; Taylor, R.; Fern, L.; Bryant-Lukosius, D.; Barr, R.; Fraser, G.; Klassen, A. Identifying the Supportive Care Needs of Adolescent and Young Adult Survivors of Cancer: A Qualitative Analysis and Systematic Literature Review. Support Care Cancer **2014**, *22*, 947–959.

[26] Frederick, N.N.; Revette, A.; Michaud, A.; Bober, S.L. A Qualitative Study of Sexual and Reproductive Health Communication with Adolescent and Young Adult Oncology Patients. Pediatric blood & cancer **2019**, *66*, e27673–n/a.

[27] Martins, A.; Taylor, R.M.; Lobel, B.; McCann, B.; Soanes, L.; Whelan, J.S.; Fern, L.A. Sex, Body Image and Relationships- Information and Support Needs of Adolescents and Young Adults: BRIGHTLIGHT. Journal of Adolescent and Young Adult Oncology **2018**, https://doi.org/10.1089/jayao.2018.0025.

[28] National Cancer Control Programme. Framework for the Care and Support of Adolescents and Young Adults (AYA) with Cancer in Ireland (2021-2026).

[29] MSN for Children and Young People with Cancer. Collaborative and Compassionate Cancer Care. **2021**.

[30] Department of Health. Regional Standards of Care for AYA in Northern Ireland. **2025**.

[31] NHS England. *The NHS Long Term Plan.*; NHS England: London, 2019.

[32] Fern, L.A.; Lewandowski, J.; Coxon, K.M.; Whelan, J.S. Available, Accessible, Aware, Appropriate and Acceptable: A Strategy for Improving Participation of Teenagers and Young Adults in Cancer Clinical Trials. Lancet Oncology **2014**, *15*, e341–e350.

[33] Parsons, H.M.; Harlan, L.C.; Seibel, N.L.; Stevens, J.L.; Keegan, T.H.M. Clinical Trial Participation and Time to Treatment among Adolescents and Young Adults with Cancer: Does Age at Diagnosis Or Insurance make a Difference? Journal of clinical oncology **2011**, *29*, 4045–4053.

[34] Fern, L.; Davies, S.; Eden, T.; Feltbower, R.; Grant, R.; Hawkins, M.; Lewis, I.; Loucaides, E.; Rowntree, C.; Stenning, S. *et al.* Rates of Inclusion of Teenagers and Young Adults in England into National Cancer Research Network Clinical Trials: Report from the National Cancer Research Institute (NCRI) Teenage and Young Adult Clinical Studies Development Group. Br. J. Cancer **2008**, *99*, 1967–1974.

[35] Bleyer, W.A.; Tejeda, H.; Murphy, S.B.; Robison, L.L.; Ross, J.A.; Pollock, B.H.; Severson, R.K.; Brawley, O.W.; Smith, M.A.; Ungerleider, R.S. National Cancer Clinical Trials: Children have Equal Access; Adolescents do Not. Journal of adolescent health **1997**, *21*, 366–373.

[36] Barr, R.D. Planning a Comprehensive Program in Adolescent and Young Adult Oncology-A Collision with Reality. Journal of Adolescent & Young Adult Oncology **2016**, *5*, 303–309.

[37] Freyer, D.R.; Seibel, N.L. The Clinical Trials Gap for Adolescents and Young Adults with Cancer: Recent Progress and Conceptual Framework for Continued Research. Curr Pediatr Rep **2015**, *3*, 137–145.

[38] Independent Cancer Taskforce. Achieving World-Class Cancer Outcomes: A Strategy for England. **2016**.

[39] Bautista-Gonzalez, E.; Taylor, R.M.; Fern, L.A.; Barber, J.A.; Cargill, J.; Dobrogowska, R.; Feltbower, R.G.; Haddad, L.; Hall, N.; Lawal, M. *et al.* Exploring the Coordination of Cancer Care for Teenagers and Young Adults in England and Wales: BRIGHTLIGHT_2021 Rapid Qualitative Study.

[40] Elsbernd, A.; Boisen, K.A.; Hjerming, M.; Niemann, C.U.; Petersen, G.; Pappot, H.; Hjalgrim, L.L. Developing Age-Appropriate Supportive Facilities, Resources, and Activities for Adolescents and Young Adults with Cancer Across Departments and Diagnoses: A Single-Center Experience. Journal of adolescent and young adult oncology **2019**, *8*, 98–102.

[41] Ferrari, A. The Challenge of Access to Care for Adolescents with Cancer in Italy: National and Local Pediatric Oncology Programs. International Perspectives on AYAO, Part 2. Journal of adolescent and young adult oncology **2013**, *2*, 112–117.

[42] Osborn, M.; Johnson, R.; Thompson, K.; Anazodo, A.; Albritton, K.; Ferrari, A.; Stark, D. Models of Care for Adolescent and Young Adult Cancer Programs. Pediatric blood & cancer **2019**, *66*, e27991–n/a.

[43] Gorin, S.S.; Haggstrom, D.; Han, P.K.J.; Fairfield, K.M.; Krebs, P.; Clauser, S.B. Cancer Care Coordination: A Systematic Review and Meta-Analysis of Over 30 Years of Empirical Studies. ann. behav. med **2017**, *51*, 532–546.
